# Strongyloidiasis: Risk and Healthcare Access for Latin American Immigrants Living in the United States

**DOI:** 10.1007/s40475-016-0065-3

**Published:** 2016-02-03

**Authors:** Graciela Ostera, James Blum

**Affiliations:** Department of Microbiology and Immunology, Georgetown University Medical Center, Washington, DC 20007 USA

**Keywords:** *Strongyloides stercoralis*, Prevalence, Immigrants, Healthcare access

## Abstract

This commentary discusses our current understanding about *Strongyloides stercoralis* prevalence rates in the United States (USA) in the context of healthcare delivery to immigrants from Latin America. A literature search reveals that while prevalence rates in Latin American countries are not well documented, the rates in Latin American immigrants living in the USA are even less well known. This limited understanding compounds the health challenge facing immigrants who already have limited access to the healthcare system. To better address this problem, we suggest improved parasitological screening plus additional funding for free clinics.

## Introduction

Large migratory waves have always shaped the ethnic composition of the United States (USA). In particular, the past 50 years have been characterized by a steady stream of immigrants from Latin America, making individuals of Hispanic/Latino origin the largest group of foreign-born in this country.

Institutions which provide healthcare services to immigrant groups need to be aware of the possible endemic conditions present in this population in order to provide adequate care and anticipate future needs.

There are almost 12 million unauthorized migrants currently living in the USA, of which 8.1 million (71 %) originated in Mexico and Central American countries, where gastrointestinal parasites are endemic due to inadequate sanitary conditions [1]. Unauthorized immigrants bypass the mandated medical examination that US immigration authorities impose on those who petition their residency. While all other immigrants who petition for legal residence in the USA must undergo some form of medical examination, only refugees receive treatment for presumptive strongyloidiasis, schistosomiasis, and soil-transmitted helminths (STH) [2].

This is of particular concern when we consider *Strongyloides stercoralis*, a nematode generally classified separately from the group of STH composed by *Ascaris lumbricoides*, *Trichuris trichiura*, *Necator americanus*, and *Ancylostoma duodenale* [3].

*S. stercoralis* causes a gastrointestinal infection that has several distinctive features. First, *S. stercoralis* can cause a lifelong infection due to its capability to re-infect its human host. This infection can become life-threatening under some conditions. Second, laboratory tests to diagnose most STH are not sensitive enough to reliably detect *S. stercoralis* due to its low larval content in stool. Third, *S. stercoralis* prevalence in Latin American countries is incomplete. Finally, the anti-helminthic drugs most frequently used in STH deworming campaigns in the region (albendazole and mebendazole) are highly effective against STHs, but they are not the preferred drugs for *S. stercoralis.*

In this commentary, we will focus on *S. stercoralis* prevalence in Central America, particularly in Mexico, Guatemala, El Salvador, and Honduras, because these are the top four Latin American countries of origin for unauthorized immigrants to the USA [1].

We will also analyze prevalence data from Latin American immigrants currently living in the USA. Though this article is not an exhaustive review on this topic, our intention is to emphasize what we know about *S. stercoralis* prevalence rates to the medical community providing health services to immigrant groups. To that end, we also discuss the medical capabilities of two large charity healthcare organizations serving predominantly Hispanic/Latino immigrants in the Washington, DC metropolitan area.

## Strongyloidiasis Prevalence in the Americas

We consulted epidemiological information made available by the Pan-American Health Organization (PAHO). PAHO plays a significant role in the systematic surveillance, design, and execution of deworming campaigns targeting STHs in the region. However, these surveillance programs have not included, so far, an assessment of *S. stercoralis* epidemiology. According to PAHO executive staff, efforts are currently underway to design and implement a much-needed systematic and standardized surveillance of *S. stercoralis* prevalence. In their literature review from July 2013, Schär and colleagues summarized the results of independent, Latin American-based studies investigating *S. stercoralis* prevalence rates. The authors note significant variations in the available data based on the type of study they found (e.g., community-based vs. hospital-based studies) as well as on the different sensitivity (low, medium, or high) of the diagnostic methods [3–5].

As an example, some *S. stercoralis* prevalence rates for selected countries are Mexico (1.6 and 5.7 %), Guatemala (unavailable and 2 %), and Honduras (3.2 and 29.8 %) for community-based and hospital-based studies, respectively [3]. Those values include diagnostic methods with different sensitivities. No data was available for El Salvador.

Collectively, this prevalence data show how difficult it would be to estimate *S. stercoralis* health risk in this sub-group of Latin American countries with the information that is currently available and underscores the need for a systematic surveillance approach.

Looking at the *S. stercoralis* data currently available on foreign-born individuals living in the USA, the Centers for Disease Control and Prevention (CDC) report a prevalence range between 0 and 46.1 % [6]. This wide range comprises data obtained from different international immigrant groups. Values on the upper end of this range correspond to studies investigating refugee groups from Africa and Asia (e.g., 46 % prevalence in a group of Sudanese refugees) [7].

We searched for *S. stercoralis* prevalence rates, limiting the query to studies performed in the USA between 1990 and 2015 on subjects born in Latin America who are not immunocompromised, and found only four studies. However, only two of these studies were performed in *healthy* individuals while the other two investigated *S. stercoralis* in patients with a medical condition [8–11] (Table 1).

**Table 1:** *S. stercoralis* prevalence studies performed in the USA on Latin American immigrants, 1990–2015

Citation	Diagnostic method	Prevalence (%)	Immigrant’s country of origin
Rapoport et al. [10]	ELISA	5.8	Brazil
Fitzpatrick et al. [8]	ELISA	4.6^a^	Mexico, Ecuador, Peru
Wehner et al. [9]	ELISA	10.5^b^	Mexico, Central and South America
Ciesielski et al. [11]	Stool sample	6.1	Mexico, Central America, and Haiti

^a^Kidney transplant candidates

^b^Patients with asthma

Clearly, prevalence values ranging from 4.6 to 10.5 % are on the lower end of the ones reported by the CDC. Recent *S. stercoralis* prevalence data obtained by our group also falls within this range (*unpublished data*). It is not known whether patients included in these studies were subjected to presumptive deworming treatments in their country of origin or in the USA that could have reduced the proportion of positive cases detected. Future studies controlling this variable are needed.

From this data, we conclude that while it may not be possible to accurately estimate the *S. stercoralis* health risk in Latin American immigrants, limited surveillance studies performed in the USA seem to indicate prevalence values between 5.0 and 10.0 %. Although actual *S. stercoralis* prevalence needs to be refined, this information can still be useful to medical service providers.

## Healthcare Options for the New and Undocumented Immigrant

In the USA, recent documented and undocumented immigrants have limited access to health care. Under the Affordable Care Act (ACA), immigrants who are lawfully present in the USA who do not have employer-provided health insurance are able to purchase private health insurance through the government’s marketplaces only after 5 years of residence in the country [12].

The vast majority of undocumented immigrants or those with a legal immigration status but who don’t have health insurance are forced to seek care through emergency rooms, federally qualified health centers (FQHC), and charity-supported free clinics, with the exception of a small number of states that use their own funds to provide Medicaid and Children’s Health Insurance Program (CHIP) coverage.

We interviewed administrative officers at two large primary care organizations that provide medical services in the Washington, DC metropolitan area to a population that is predominantly uninsured or below the federal poverty line. Combined, these two organizations provide over 70,000 patient visits per year. Eighty percent of these services are delivered to uninsured individuals of Hispanic origin.

In 2014, the smaller of these two organizations provided medical care worth 4.7 million US dollars to individuals who were unable to pay for medical services.

Maria Gomez, the president and CEO of Mary’s Center, a FQHC in Washington, DC stressed that these clinics deliver much-needed primary care in response to top medical concerns in the adult and pediatric populations they serve. Services focus on frequently observed conditions such as obesity, hypertension, diabetes, prenatal care, and several common pediatric concerns. However, these two healthcare organizations stated that they are not able to offer routine parasitological screening to their patients due to the high cost of laboratory tests. One clinic admitted that specific parasitological training would be beneficial to increase awareness in their team of primary care practitioners (doctors and nurses). Unfortunately, *S. stercoralis* infection can have fairly unspecific symptoms and practitioners may not consider this presumptive diagnosis initially.

## Conclusion

Our current understanding of *S. stercoralis* infection prevalence rates in Latin American immigrants in the USA is affected by the following factors:

1. Lack of standardized prevalence data available from countries in the Latin American region
2. Very limited number of studies documenting *S. stercoralis* prevalence rates in Latin American immigrants living in the USA
3. Limited healthcare accessibility for immigrants: generally limited to primary care, which may be affected by cost restrictions on diagnostic services for gastrointestinal parasitic infections

Given the current global landscape, it is critical that we are prepared to identify and treat patients with neglected parasitic infections (NPI) coming to the USA from different parts of the world. We should consider the systematic screening of parasitic diseases by immigration authorities at borders and community-based screening in areas with high immigrant density. For clinics that are already providing health care to vulnerable populations, additional funding is needed to cope with an increased number of patients and complexity of services.
